# Female adolescent health needs and determinants

**DOI:** 10.5935/1518-0557.20200011

**Published:** 2020

**Authors:** Sepideh Panjalipour, Zahra Bostani Khalesi, Sedighe Rezaie-Chamani, Ehsan Kazemnejad

**Affiliations:** Social Determinants of Health Research Center, Guilan University of Medical Sciences, Rasht, Iran.

**Keywords:** female adolescents, healthcare needs, needs assessment

## Abstract

**Objective:**

Adolescence is a critical developmental period with characteristic health risks and needs. Assessing adolescent health needs helps to improve the planning and implementation of effective interventions. This study aimed to describe the health needs and determinants of female adolescents.

**Methods:**

This analytical descriptive study included 850 female students. The sampling method was multistage cluster sampling. Data were collected from a questionnaire consisting of two parts, the first probing into socio-demographic matters and the second into respondent health needs. Data analysis was performed via the Mann-Whitney and Kruskal-Wallis tests, and Mann-Whitney, Kruskal-Wallis, and Pearson correlation coefficients.

**Results:**

The results showed that individuals attending private high schools not living with their parents had higher health needs than other students. Students with older fathers - particularly fathers aged 50+ years - had increased health needs. Psycho-emotional care topped the list of health needs, whereas services in the field of spiritual belief were in last place.

**Conclusion:**

Our study found that the most important factors related to healthcare needs were level of education, type of school (private *vs.* public), and living with parents. The results of this study can be useful in designing and implementing interventions to prevent high-risk behaviors and promote adolescent health.

## INTRODUCTION

Adolescence is a vital and very important stage of life, a passage from childhood to adulthood that marks the beginning of physical, psychological, and social transformation (Dick & Ferguson, 2015). Adolescence includes individuals aged 10-19 years (Melaku *et al*., 2015). Adolescents make up more than a fifth of the world’s population (Taghizadeh Moghaddam *et al*., 2016). They account for a significant portion of our nation’s population and their health deserves careful consideration (Panjalipour *et al*., 2018). The characteristics of female puberty, the physical and psychological challenges of adolescence, and the vital role of female infertility add up to reinforce the relevance of female adolescent health. Adolescence is the basis from which women form their later lives and has direct impact on their future families and children (Jaskiewicz, 2009). The first step in designing and planning care strategies is assessing the needs of the target groups. At the International Conference on Population and Development, emphasis was placed on the difference between the needs of adults and adolescents. Participating nations were asked to identify and address these needs (Chandra-Mouli *et al*., 2015). Despite the differences inherent to adolescent populations, few studies have been conducted to assess their specific health needs.

A review showed that studies looking into the health needs of female adolescents tend to focus on building awareness and informing readers about puberty, leaving out the physical, mental, social, and educational dimensions of health as defined by the World Health Organization (Golchin *et al*., 2012). A study on puberty suggested there is a significant need for sexual health education among adolescents (Emami *et al*., 2007). Other studies reported on the incidence of mental disorders in adolescents (Khalajabadi-Farahani, 2015; Simbar *et al*., 2017). The study by Simbar also showed that sexual education among adolescents is necessary. Unfortunately, because of shame, taboo, and cultural beliefs in the community, proper education about reproduction for adolescents provided by their families and schools and the inclusion of the subject in textbooks have been ignored (Shahhosseini *et al*., 2013).

This study analyzed female adolescent health needs and determinants.

## MATERIALS AND METHODS

This analytical descriptive study looked into the health needs of female adolescents and related factors. Female students aged 10-19 years attending private and public high schools were included. Participants were chosen based on a random multistage sampling method.

The data used in our study were collected from a two-part questionnaire. The first featured 18 validated questions covering socio-demographic factors (age, grade, field of study, place of residence, ethnicity, number of family members, parent ages, child grade in the family, parental schooling, parental occupation, parental income, living with parents, having disorders) designed by the author to evaluate health needs and related factors. The second part was the Female Adolescent Health Needs Questionnaire (FAHNQ). The FAHNQ includes 65 questions in five domains, namely: physical (14 items); emotional (16 items); social (16 items); education (14 items); and spiritual (5 items). The FAHNQ is a 5-point Likert scale developed by Shahhosseini *et al*. (2012a) to assess the health needs of female adolescents in current and desired conditions.

The face validity of the questionnaire was tested with 30 females. Content validity was rated at 92%. The reliability of the questionnaire was assigned a Cronbach’s alpha of 0.90. Participants were asked to define their needs and rate them based on the distance between the present and desired situation in a 5-point Likert scale ranging from 1 (completely disagree) to 5 (completely agree). The scores in the current and desired conditions were calculated as percentages.

Statistical analysis was performed with descriptive and analytical methods on SPSS version 21. Statistical indices such as mean, standard deviation, median, minimum and maximum were used to describe the health needs of female adolescents. Comparisons were made based on independent t-test and ANOVA findings. Regression methods were used to derive the factors related to the health needs of female adolescents and to find whether the assumptions of non-parametric tests were not met. The Mann-Whitney and Kruskal-Wallis tests were used. Statistical significance was attributed to events with a *p*<0.05.

## RESULTS

The mean age of participants was 15.3±1.6 years. The youngest was 12 and the oldest was 18 years old. The mean age of the fathers of the participants was 45.2±6.2 years; the mean age of their mothers was 40.7±5.7 years (Table 1).

**Table 1:** Frequency distribution of participants according to demographic characteristics

Variables		Number (individuals)	Percent (%)
Education center	Public	663	78
	Private	187	22
Study field	Math	80	9.4
	Experimental	185	21.8
	Humanities	153	18
	Vocational	63	7.4
	First .m	369	43.4
Area or residence	City	816	96
	Suburbs	34	4
Ethnicity	Guilanian	732	86.1
	Other	118	13.9
Number of family members	2	21	2.5
	3	200	23.5
	4	466	54.8
	5	120	14.1
	6- and more	4	5.1
Child's grade	1	485	57.1
	2	240	28.2
	3	90	10.6
	4- to up	35	4.1
Father's level of education	Illiterate	15	1.8
	Primary	95	11.2
	Middle school degree	265	31.2
	Diploma	363	42.7
	University educated	112	13.2
Father's job	Unemployed	16	1.9
	Worker	79	9.3
	Farmer	17	2
	Clerk	198	23.3
	Self-employed	540	63.5
Mother's level of education	Illiterate	16	1.9
	Primary	102	12
	Middle school degree	258	30.4
	Diploma	394	46.4
	University educated	80	9.4
Mother's job	Housewife	731	86
	Worker and farmer	10	1.2
	Clerk	40	4.7
	Self-employed	69	8.1
Family income	Less than 1 million	213	25.1
	1- 2 million	432	50.8
	More than 2 million	205	24.1
Living with parents	Yes	803	94.5
	No	47	5.5

The mean health need scores were as follows: physical domain (15.1%±10.3%); emotional (23.5%±2%); social (21.1%±11.7%); education (18.6%±16.1%); spiritual (11.9%±13.9%); and overall health need score (18.8%±10.1%). The highest mean score occurred in the emotional domain (23.47%), and the lowest in the spiritual domain (11.99%) (Table 2).

**Table 2:** Mean scores of participants' health needs

Health needs	Mean	SD	Average	Min	Max	Confidence interval 95%	
						Min	Max
Physical	15.12	10.34	14.75	0	48.48	14.42	15.82
Emotional	23.47	12.22	25	0	59.09	22.64	24.29
Social	21.02	11.68	21.25	0	58.75	20.23	21.80
Education	18.63	16.01	15.71	0	72.31	17.55	19.71
Spiritual	11.99	13.86	8	0	73.68	11.06	12.93
Total	18.80	10.10	18.57	0	51.93	18.12	19.48

Results for some variables revealed that attending private school (*p*=0.012), living without the parents (*p*=0.018), and father’s age (*p*<0.001) were significantly related with having higher health needs. Participants attending private compared to public schools and individuals not living with their parents had more health needs. Father's age increased in line with health needs (Table 3).

**Table 3:** Regression coefficient of factors related to health needs

Variable	Regression coefficient	SD	Confidence interval 95%		Test statistics	Significance level
			Min			
Comparison	9.362	1.086	7.230	8.618	0.001	0.001
Second compared to first grade	6.070	0.665	4.764	9.123	0.001	0.001
Comparison	7.080	1.446	4.241	4.894	0.001	0.001
Private compared to public	1.896	0.796	0.334	2.382	0.017	0.017
Comparison	3.609	2.142	-0.595	1.685	0.092	0.092
Private compared to public	1.968	0.795	18.57	2.476	0.130	0.130
Do you live with your parents?	3.160	1.441	0.408	2/193	0.029	0.029
Comparison	-1.450	3.207	-7.744	-0.452	0.651	0.651
Second, compared to first grade	5.815	0.673	4.495	8.644	0.001	0.001
Private compared to public	2.009	0.794	0.451	2.531	0.012	0.012
Not living with parents	3.413	1.443	0.581	2.365	0.018	0.018
Father's age	0.114	0.054	0.001	2.117	2.117	0.035

## DISCUSSION

The results presented in this study suggest that the emotional domain sits atop the list of health needs of female adolescents. This finding is in line with Shahhosseini *et al*. (2012b). Amin (2015) suggested that mental health is a significant challenge for adolescents globally. Esmaeili & Rabiei (2008) reported that female adolescents prioritize their‏ emotional needs, while parents focus on their children’s physical needs. Since school plays an important role in the self-esteem and satisfaction with life of adolescents, reforms of the educational system looking at the needs of adolescents might be extremely valuable (Townsend & Bates, 2007). Somewhat surprisingly, we found that social needs had a secondary role. A qualitative study on the health needs of female adolescents from Mazandaran found that family was a highly ranked element (Shahhosseini *et al*., 2012b). Other authors indicated that the lack of good family relationships might increase the chances of adolescents engaging in hazardous behaviors such smoking (Dick *et al*., 2007). Having a good relationship with a supportive family increases self-esteem, life satisfaction, the ability to cope with stressful situations, and reduces depression (Milevsky *et al*., 2007; Oliva *et al*., 2009). Educational needs ranked third from the viewpoint of participants in this study.

The results of Simbar *et al*. (2017) revealed that sexual education is a significant need among female adolescents. However, issues such as shame, taboo, and cultural beliefs have impeded the administration of proper reproductive health education for adolescents in family and school settings. The results of this study were consistent with the findings reported by Bazarganipour *et al*. (2013), which indicated that high school students felt the need for sexual education. Studies in this field performed in Iran revealed a knowledge gap on puberty and sexual health among adolescents (Nejad *et al*., 2012; Koohestani *et al*., 2009). The reproductive health needs of adolescents have gained attention in recent international conferences on population and development (Olfati & Aligholi, 2008). The reproductive health of adolescents has become a topic of global interest (Ghahremani *et al*., 2014).

Quick physical growth and development characteristically seen in adolescence lead to increased calorie and nutritional requirements. Therefore, proper nutrition may affect growth and puberty development (Jodhun *et al*., 2016). Iranian adolescents appear to have less than recommended quantities of certain foods (Koochakpour *et al*., 2012). Adolescents who do not have a proper breakfast learn less at school. Snacks play a very important role in energy supply and are often more important than the main meals (Karami & Ghaleh, 2015). The need ranked fifth by participating adolescents was belief. In the belief domain, the highest need was “deductive rituals.” Beliefs easily accepted in childhood are questioned in adolescence. Teenagers seek rituals based on their logic and wisdom (Sajjadi *et al*., 2012). This issue requires attention. Neglecting it may result in irreparable outcomes.

Our study indicated a relevant association between female adolescent health needs and place of education, living with parents, and father's age. Participants attending private schools had more health needs than individuals going to public schools. According to Marlow's theory, people with met basic needs have demands concerning higher level needs (Gawel, 1997). Our study indicated that participants not living with their parents due to divorce or death of one of the parents had increased health needs. Fomby & Cherlin (2007) suggested that family is the smallest social unit, which stability affects all its members. Children of divorced parents have different needs than children of married parents. We found‏ an inverse correlation between father age and participant health needs. Individuals with fathers aged 50+ years had more health needs than subjects with fathers aged less than 50. The results of Afshary *et al*. (2016) indicated a relevant association between father age and the health need of female adolescents.

## CONCLUSION

This study was in line with previous studies and emphasized the health needs of adolescents. Neglecting health needs may result in irreparable consequences for the next generation. The studies cited above assessed the needs of adolescents based on instruments developed by their authors, while our study used mental assessment instruments to better define the needs and the distance between the current and the desired condition, with the purpose of producing more scientific results. Previous studies have performed one-dimensional analyses of needs, while ours assessed all dimensions of health needs. The health needs of male and female adolescents in other Iranian ethnicities and cultures, adolescents with special needs, and working adolescents should be considered in future studies. The present study had important limitations. Participant mental conditions may have affected the answers.
